# Discovery of a Novel Natural Allosteric Inhibitor That Targets NDM-1 Against *Escherichia coli*

**DOI:** 10.3389/fphar.2020.581001

**Published:** 2020-10-02

**Authors:** Yanan Yang, Yan Guo, Yonglin Zhou, Yawen Gao, Xiyan Wang, Jianfeng Wang, Xiaodi Niu

**Affiliations:** ^1^College of Food Science and Engineering, Jilin University, Changchun, China; ^2^Key Laboratory of Zoonosis Research, Ministry of Education, College of Veterinary Medicine, Institute of Zoonosis, Jilin University, Changchun, China

**Keywords:** metallo-β-lactamases, carnosic acid, molecular modeling, allosteric inhibitor, *Escherichia coli*

## Abstract

At present, the resistance of New Delhi metallo-β-lactamase-1 (NDM-1) to carbapenems and cephalosporins, one of the mechanisms of bacterial resistance against β-lactam antibiotics, poses a threat to human health. In this work, based on the virtual ligand screen method, we found that carnosic acid^1^ (CA), a natural compound, exhibited a significant inhibitory effect against NDM-1 (IC_50_ = 27.07 μM). Although carnosic acid did not display direct antibacterial activity, the combination of carnosic acid and meropenem still showed bactericidal activity after the loss of bactericidal effect of meropenem. The experimental results showed that carnosic acid can enhance the antibacterial activity of meropenem against *Escherichia coli* ZC-YN3. To explore the inhibitory mechanism of carnosic acid against NDM-1, we performed the molecular dynamics simulation and binding energy calculation for the NDM-1-CA complex system. Notably, the 3D structure of the complex obtained from molecular modeling indicates that the binding region of carnosic acid with NDM-1 was not situated in the active region of protein. Due to binding to the allosteric pocket of carnosic acid, the active region conformation of NDM-1 was observed to have been altered. The distance from the active center of the NDM-1-CA complex was larger than that of the free protein, leading to loss of activity. Then, the mutation experiments showed that carnosic acid had lower inhibitory activity against mutated protein than wild-type proteins. Fluorescence experiments verified the results reported above. Thus, our data indicate that carnosic acid is a potential NDM-1 inhibitor and is a promising drug for the treatment of NDM-1 producing pathogens.

## Introduction

The inhibition of bacterial infection has important implications for human health (al Jalali and Zeitlinger, 2018; Li et al., 2018; Shamriz and Shoenfeld, 2018). At present, β-lactam antibiotics, including penicillins (Liu et al., 2018a), carbapenems (Edwards and Betts, 2000), and cephalosporins (Paterson et al., 2001), have been widely used to treat bacterial infections (Essack, 2001). However, extensive use of antibiotics has led to the continued spread of drug-resistance (Khan et al., 2017a). Widespread resistance mechanisms of bacteria have lowered the effectiveness of antibiotics, leading to a health crisis (Khan et al., 2017b).

In 2008, the enzyme New Delhi metallo-β-lactmase-1 (NDM-1) was first reported (Groundwater et al., 2016). It was isolated from *Klebsiella pneumoniae* in India and belonged to the B1 subclass MBLs (Brem et al., 2016). Currently, bacteria containing the NDM-1 gene are found in many regions (Johnson and Woodford, 2013; Tran et al., 2015; Heinz et al., 2019). NDM-1 has been shown to be resistant to β-lactamase inhibitors mainly due to its ability to hydrolyze the amide bond of β-lactam ring (King et al., 2012). Moreover, the resistance of pathogenic bacteria has created a great challenge to the treatment of diseases (Ejaz et al., 2020). Therefore, NDM-1 can be targeted to inhibit the resistance of pathogenic bacteria.

At present, NDM-1 is often found in *Escherichia coli* (Zhang et al., 2017*)*. Various pathogenic *E. coli* strains can cause diseases such as human diarrhea (Shah et al., 2015), urinary infections, and blood infections (Tuem et al., 2018). In recent years, *E. coli* resistance in the population has increased (Wang X. Q. et al., 2015). In addition, the resistance of bacteria to antibiotics increases medical costs and threatens human health (Yang et al., 2017). NDM-1 plays an important part in the resistance mechanism of bacteria (Du et al., 2017). At present, *E. coli* producing NDM-1 have been found in many countries (Erb et al., 2007) and has been known to continuously spread (Nordmann et al., 2012). At the same time, the plasmid with blaNDM-1 in the patient can spread among different *Enterobacteriaceae* species (Martino et al., 2019). Therefore, it is important to find an effective drug that inhibits the resistance of NDM-1 producing *E. coli*.

Recently, several inhibitors of NDM-1 have been discovered and synthesized. Among the currently found inhibitors, captopril and its analogs may be the desirable candidates. Among them, the IC_50_ values of d- and l-captopril for inhibiting NDM-1 in the hydrolysis of imipenem were 7.9 and 202.0 μM, respectively. However, they had no effect on bacteria carrying carbapenemases (Chiou et al., 2015). Jian Zhang et al. found that Aspergillomarasmine A derivatives can inhibit NDM-1 and overcome antibiotic resistance (Zhang et al., 2017). Scott J. Hecker *et al*. found that cyclic boronic acid QPX7728 showed effective activity on β-lactamases including NDM-1 (Hecker et al., 2020). It also showed similar pharmacokinetics to β-lactam antibiotics in rats and had good oral bioavailability. In addition, Chen Cheng et al. found that disulfiram is a promising candidate for the development of NDM-1 inhibitors, which can covalently bind to NDM-1 by forming an S–S bond with Cys208 residue at the active site (Chen et al., 2020b). At the same time, some metal complexes (cisplatin and Palladium(II) complexes) (Chen et al., 2020a) and DNA aptamers (Khan et al., 2019) have also been found to have inhibitory effects on NDM-1. Among them, the metal complexes inhibited MβLs through a new inhibition mode, which binds to the Cys208 residue in the active site, causing Zn ^2+^ to be replaced by other ions. Moreover, scholars have also discovered some natural inhibitors. Xuequan Wang *et al*. purchased 44 compounds through virtual screening and found three new NDM-1 inhibitors with smaller IC_50_ values (Wang X. Q. et al., 2015). The most effective inhibitor displayed an IC_50_ value of 29.6 ± 1.3 μM. However, in these previous literatures, most of these inhibitors may be competitive inhibitors, and their binding regions are all located in the active sites of NDM-1, which can easily lead to multiple drug resistance. Consequently, the demand for novel non-competitive inhibitors of NDM-1 is increasing.

In our work, a natural compound carnosic acid (CA) was identified as an effective inhibitor against the bioactivity of NDM-1 using virtual screening. Molecular dynamics simulations and binding free energy calculations further revealed that the bioactivity of NDM-1 was effectively inhibited due to the binding of carnosic acid with the allosteric pocket. Furthermore, it was identified that carnosic acid can significantly increase the antibacterial activity of meropenem. These results indicate that carnosic acid has the potential to become the novel NDM-1 inhibitor.

## Materials and Methods

### Bacterial Strains and Chemicals

The isopropyl β-d-thiogalactoside (IPTG) and kanamycin were purchased from Dalian Meilun Company (Dalian, China). The carnosic acid (≥98% pure), meropenem (≥87% pure) and other chemicals were obtained from the National Institutes for Food and Drug Control (Beijing, China). Dimethyl sulfoxide (DMSO) was purchased from Sigma-Aldrich (St. Louis, MO, USA). The bacterial strains used in this study are showed in **Table 1**.

**Table 1:** The bacterial strains list.

Strain	Relevant genotype	Source or Reference
*E. coli* BL21	Expression strain	Invitrogen
*E. coli* ZC-YN3	producing NDM-1	(Liu et al., 2018b)

### Plasmid Construction of blaNDM-1 and Its Mutant

The coding sequence of the NDM-1 gene were amplified from genomic DNA of *E. coli* and digested with BamHI and XhoI. Subsequently, it was cloned into pET28a to generate plasmid pET28a-NDM-1. Plasmids encoding F46A-NDM-1, L65A-NDM-1, and T94A-NDM-1 were constructed using a QuikChange site-directed mutagenesis kit. The ligation product was transformed into competent *E. coli* DH5α cells. All constructed strains were verified by PCR and DNA sequencing (Ali et al., 2018). The primer pairs used in the work are listed in **Table 2**. The NDM-1 primer came from the literature (Teng et al., 2019), and the other primers were designed in this experiment.

**Table 2:** Oligonucleotide primers used in this study.

Primer name	Oligonucleotide (5′–3′)
NDM-1-F	GCGCGGATCCGTGCTGGTGGTCGATAC
NDM-1-R	GCGCCTCGAGTCAGCGCAGCTTGTCG
F46A-F	GAAACTGGCGACCAACGGGCGGGCGATCTGGTTTTCCG
F46A-R	CGGAAAACCAGATCGCCCGCCCGTTGGTCGCCAGTTTC
L65A-F	CACACTTCCTATGCGGACATGCCGGGTTTC
L65A-R	GAAACCCGGCATGTCCGCATAGGAAGTGTG
T94A-F	GATACCGCCTGGGCGGATGACCAGAC
T94A-R	GTCTGGTCATCCGCCCAGGCGGTATC

### Protein Expression and Purification

The pET28a-NDM-1 plasmid was introduced into *E. coli* BL21 cells. The cells were cultured in the LB medium with kanamycin (50 μg/ml) at 37°C. When the cells were grown to OD_600_ = 0.6, the cells were added IPTG (1 mM final concentration) to induce protein expression and cultured overnight at 16°C. The cells were harvested by centrifugation at 10,000 rpm for 5 min and resuspended. Subsequently, the cells were sonicated and centrifuged at 10000 rpm for 1 h. The supernatant was applied to a Ni-NTA column and the non-specific binding proteins were removed with buffer (20 mM imidazole, Tris-HCl, pH 7.4). The target protein was eluted with buffer (200 mM imidazole, Tris-HCl, pH 7.4). The purified protein was concentrated and desalted (Hinchliffe et al., 2016).

### Enzyme Inhibition Assays

According to the methodology of Liu et al. the enzyme and nitrocefin were mixed and incubated at 37°C for 10 min. Consequently, absorbance was measured at 492 nm in a microplate reader. The specific calculation method was obtained from the literature (Liu et al., 2018b).

### Determination of Minimum Inhibitory Concentration (MIC), Growth Curves, and Time-Killing Assays

The minimum inhibitory concentration (MIC) of carnosic acid to *E. coli* was determined by the broth microdilution method according to the Clinical and Laboratory Standards Institute (CLSI) guidelines (CLSI, 2019). Specifically, the strain was co-cultured with various concentrations of meropenem (0–128 μg/ml) and carnosic acid (0–128 μg/ml) at 37°C for 18 to 24 h. To plot the growth curves, overnight cultured *E. coli* was enlarged (1:50) into fresh BHI broth and incubated for different lengths of time at 37°C with or without carnosic acid. The absorbance was measured at OD_600_ (Liu et al., 2018b). The potential bactericidal effect of carnosic acid was tested using the time-killing assay. According to the literature (Lagerback et al., 2016), the bacteria were diluted to 5 × 10^5^ CFU/ml and incubated with carnosic acid and meropenem. Samples were taken at specific time to determine the bacterial count to plot the time consumption curves.

### Virtual Screening

The virtual screening was performed based on compound docking to the NDM-1 *via* Autodock vina software (Hu et al., 2015). Notably, approximately 143,758 natural compounds are available on the ZINC database (Sterling and Irwin, 2015). The ligand structure files (.sdf) downloaded in batches in the ZINC database were converted into 3D structure files (.pdbqt). The 3D structure of NDM-1 came from Protein Data Bank (PDB ID is 5JQJ), which serves as the target structure for virtual screening. The Auto Dock tools was used to add polar hydrogen atoms to NDM-1. Subsequently, a grid box was constructed as the ligand docking site (center_x = −17.96 Å, center_y = −17.588Å, and center_z = 12.27 Å; and size_x = 22 Å, size_y = 28 Å, and size_z = 20 Å). Virtual screening of the natural compounds from the ZINC database was performed *via* compound docking to NDM-1 using AutoDock Vina software. The entire virtual screening calculation process used the Lamarckian (LGA) algorithm. The target protein NDM-1 was always rigid, and all twisted bonds of the inhibitor can rotate freely. The docking results were then sorted and filtered to function as the basis for the experimental investigations. The docking score for the ligands is the binding energy of compounds with NDM-1.

### Molecular Docking

The structure of NDM-1 was derived from the Protein Data Bank with PDB encoded as 5JQJ. The initial structure of the free protein was obtained using a molecular simulation of 100 ns and subsequently used for molecular docking with the ligands. The structure of carnosic acid was optimized by Gaussian 03 program. The standard docking procedures for NDM-1 and carnosic acid were performed using Auto Dock4 software. The Lamarckian genetic algorithm (LGA) was used for the docking calculations. A total of 150 independent runs were carried out with a maximum of 25,000,000 energy evaluations and a population size of 300. A grid box of dimensions (40×40×40) with a spacing of 1 Å was created and centered on the mass center of the NDM-1. The detailed docking process was referenced from previous studies (Niu et al., 2017).

### Molecular Dynamics Simulation

The molecular modeling of the interaction between NDM-1 and carnosic acid was carried out after docking as described in the above experimental method (Niu et al., 2017; Nikitina et al., 2018). The system was simulated for 100 ns using the Amber 99sb force field (Sakkiah et al., 2018) and the TIP3P water model (Lim et al., 2019). The free binding energy between NDM-1 and the ligand was calculated by the Molecular Mechanics/Generalized Born Surface Area (MM-GBSA) method (Liu et al., 2018b; Zeb et al., 2019; Nie et al., 2020).

### Fluorescence-Quenching Assay

The binding constant (*K_A_*) of the binding site of carnosic acid to wild type NDM-1 (WT-NDM-1), F46A-NDM-1, L65A-NDM-1, and T94A-NDM-1 was measured using a fluorescence quenching method. The binding energy was calculated according to the equation: *r*/*D_f_* = *nK* − *rK*. This method referred to the previous literature (Wang J. F. et al., 2015), using an excitation wavelength of 280 nm and an emission wavelength of 345 nm.

### Determination of Enzyme Reaction Rate

The principle of the measurement is mainly by using nitrocefin as an indicator, and its color changes from yellow to red with the increase of hydrolysis. NDM-1 (250 ng/ml) was incubated with various concentrations of carnosic acid in phosphate buffered saline, and then 50 μg/m L of nitrocefin was added to the mixture (Teng et al., 2019). The reaction rate was determined by continuously measuring the absorbance at OD_492 nm_ at different times (Krupyanko, 2009).

### Statistical Analysis

The statistical analysis of the results was performed using a two-tailed Student’s t-test. The difference was considered to be statistically significant when the P was less than 0.05.

## Results

### The Carnosic Acid Inhibits the Activity of NDM-1

**Figure 1:**
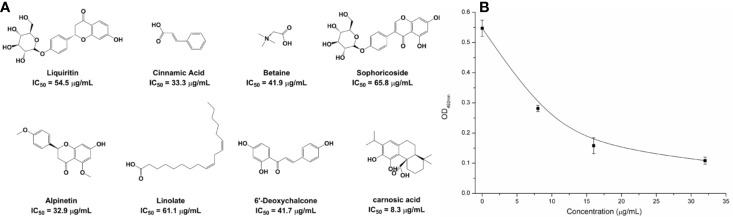
The potently inhibitors of NDM-1 based on the virtual screening approach. **(A)** Chemical structures of screening hits; **(B)** Carnosic acid inhibits the bioactivity of NDM-1.

Based on the virtual screening approach, 8 commercially available compounds were tested by the enzyme inhibition assays. The results showed that carnosic acid had a significant effect on the NDM-1 activity *in vitro* (IC_50_ = 27.07 μM) (**Figure 1A**). As is shown in **Figure 1B**, when 8 μg/ml of carnosic acid were added, the activity of the protein was seen to decrease by 48.56%, indicating that the drug significantly inhibited the protein. The protein treated with 32 μg/ml carnosic acid displayed the least activity (19.85%).

### The Carnosic Acid Potentiates Inhibitory Activity of Meropenem Against *E. coli*

**Figure 2:**
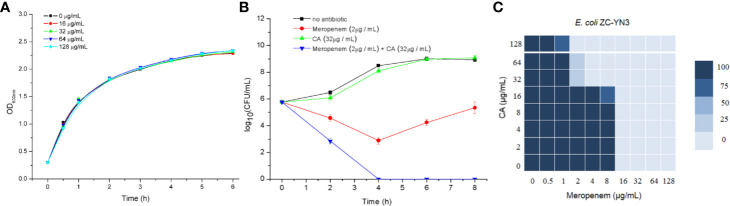
The carnosic acid potentiates meropenem inhibitory activity against *E. coli*. **(A)** Growth curves of *E. coli* ZC-YN3 cultured with different concentrations of carnosic acid; **(B)** Time-kill curves of *E. coli* ZC-YN3 with the indicated treatment. These values are the average of three independent experiments; **(C)** The microdilution checkerboard analysis showed the combined effects of carnosic acid and meropenem on *E. coli* ZC-YN3. The data are the results of four independent experiments.

It was found by MIC experiments that carnosic acid can improve the antibacterial effect of meropenem. As shown in **Table 3** and **Figure 2C**, the combination of meropenem and carnosic acid reduced the MIC of meropenem against *E. coli* ZC-YN3 (producing NDM-1) by a factor of 4 compared to meropenem alone. It was worth noting that neither an effective bacteriostatic effect (MIC > 32 μg/ml) nor inhibitory effect on the growth of *E. coli* ZC-YN3 (**Figures 2A, C**) by carnosic acid was observed in our experimental conditions.

**Table 3:** MIC (μg/ml) of meropenem against *E. coli*.

Strain	Meropenem	Combination
*E. coli* ZC-YN3 (NDM-1)	16	4

To verify the above conclusion, the time-kill curves were analyzed. The results showed that the combination of carnosic acid and meropenem was more effective than meropenem alone (**Figure 2B**). After 2 hours, the inhibitory effect of meropenem and carnosic acid on *E. coli* was stronger than that of meropenem alone. After 4 hours, the inhibitory effect of meropenem alone on *E. coli* was weakened, but meropenem and carnosic acid still had a strong inhibitory effect.

### Determination of the Binding Mode of NDM-1 With Carnosic Acid

**Figure 3:**
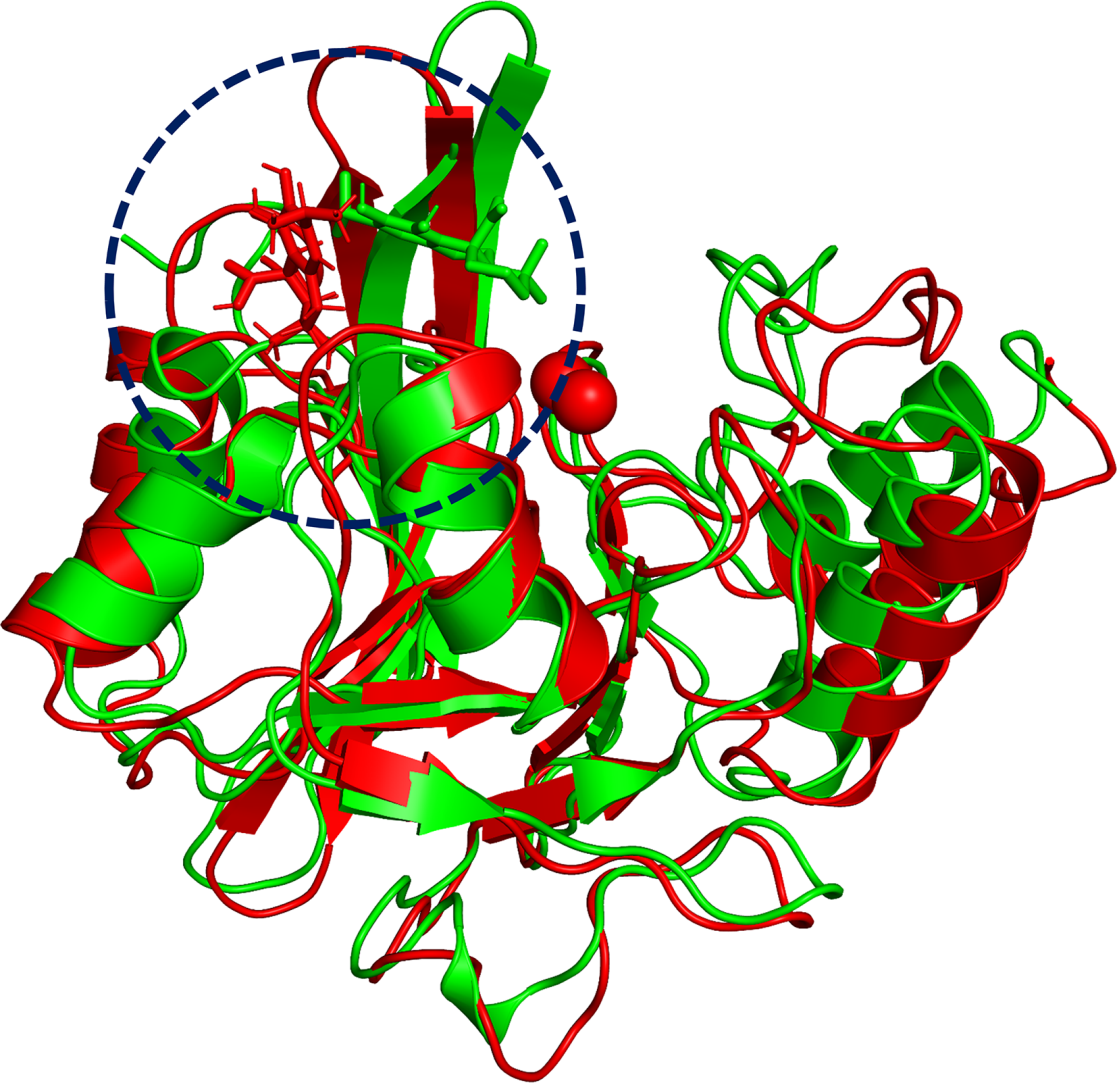
The overlap structures of complex based on molecular docking (green molecule) and MD simulation (red molecule). The binding position of ligand obtained from molecular docking is very close to the binding region of ligand obtained from MD simulation. The binding regions of complexes is within the dashed line.

**Figure 4:**
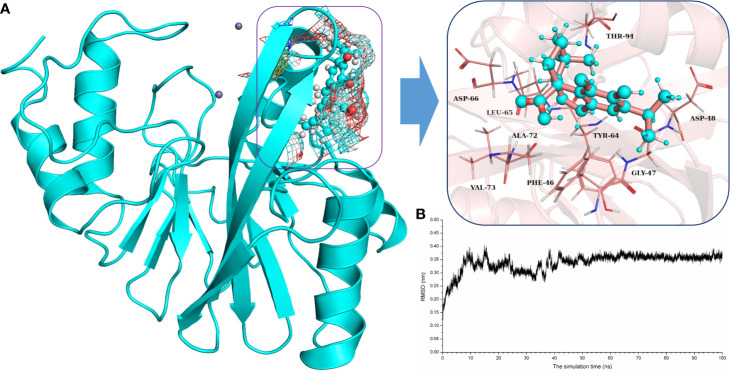
The potently binding mode of carnosic acid with NDM-1 *via* molecular modeling. **(A)** The stable 3D structure of carnosic acid with NDM-1 based on the MD simulation; **(B)** The RMSD values of NDM-1 with carnosic acid complex.

To explore the binding mechanism of NDM-1 and carnosic acid, the protein structure was docked with the drug using AutoDock 4.0 as the initial structure. The lowest energy conformation was chosen for further study. As shown in **Figure 3**, the stable binding sites of ligand with NDM-1 obtained from MD simulation were placed very near the binding position based on the molecular docking. During the 100 ns simulation, the 3D structure of NDM-1-CA was obtained (**Figure 4A**) and the root-mean-square deviation (RMSD) value of the protein backbone was calculated (**Figure 4B**). **Figure 4B** shows that the NDM-1-CA structure tended to be stable during the last 50 ns simulation. During the simulation, the main binding affinity of the carnosic acid binding to NDM-1 was hydrogen bonding and hydrophobic interactions. Specifically, the side chains of Phe46, Tyr64, Leu65, Asp66, and Thr94 can form the strong interactions with carnosic acid, as shown in the 3D structure of the complex (**Figure 4A**). Subsequently, an energy decomposition analysis was performed to confirm the above results.

**Figure 5:**
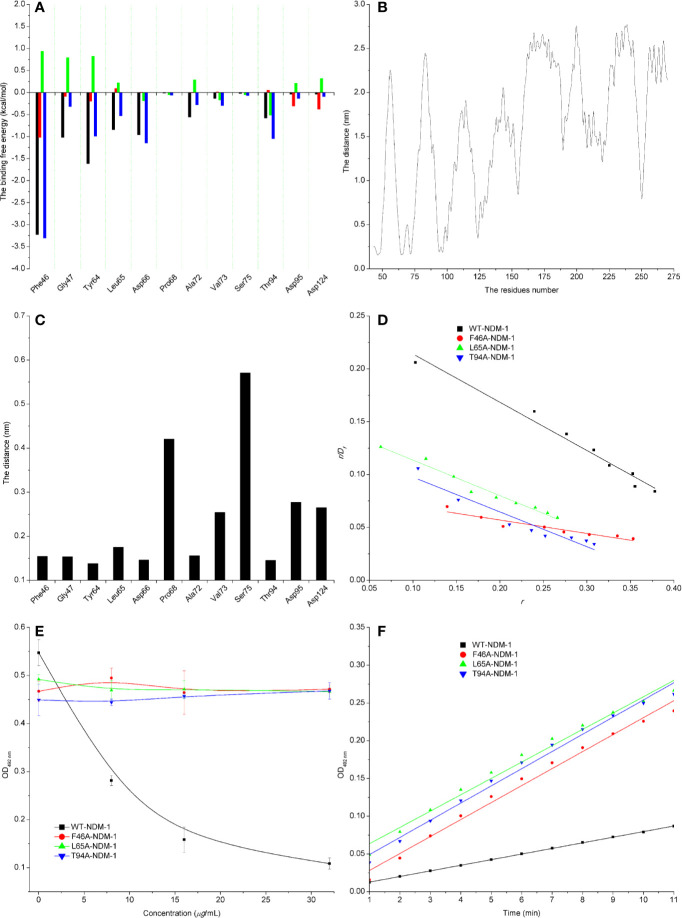
Confirmation of the binding sites of NDM-1-carnosic acid complex. **(A)** The ΔEvdw(black), ΔEele (red), ΔEsol (green), and ΔEtotal (blue) at the binding sites among NDM-1 with carnosic acid; **(B)** Analysis of the distance between all residues of NDM-1 and carnosic acid; **(C)** The distance between the NDM-1 and carnosic acid binding site residues; **(D)** The Scatchard plots of *r/D_f_* vs. *r* for carnosic acid binding to WT-NDM-1 and mutant NDM-1; **(E)** The results of enzyme inhibition assays were performed with WT-NDM-1 and mutants; **(F)** The steady-state kinetics of the hydrolysis activity of WT-NDM-1 and mutants treated with carnosic acid. The absorbances of substrate at 492 nm are used as a function of time. The slope of the fitted lines are the hydrolysis reaction rates.

The energy contribution of the selected residues was summarized in **Figure 5A**. Phe46 with a *ΔE_vdw_* of less than −0.8 kcal/mol and the Tyr64 and Asp66 residues were observed to exhibit a strong Van der Waals interaction with carnosic acid, as these residues were proximate to the carnosic acid. In addition, Phe46 was seen to provide a significant electrostatic contribution (*ΔE_ele_* ≤ −1.0 kcal/mol). At the same time, the residues Tyr64, Leu65, Asp66, and Asp94 also displayed strong Van der Waals interactions with carnosic acid (with the *ΔE_total_* = −1.0, −0.53, −1.15, and −1.05 kcal/mol, respectively). In addition, the average distances between the carnosic acid and different residues of NDM-1 during the simulation were analyzed. As shown in **Figures 5B, C**, residues Phe46, Tyr64, Leu65, Asp66, and Thr94 were closer to carnosic acid than other residues, and the distance values < 0.2 nm (The distances between different groups are the distances between the center of mass of carnosic acid and the center of mass of residues of NDM-1 in this manuscript. The distances between the center of mass of different groups are not the real distances of different atoms.). These results indicated that residues of Phe46, Tyr64, Leu65, Asp66, and Thr94 contributed to the major binding affinity for complex binding.

To detect the accuracy of the binding sites, the mutant complexes F46A-NDM-1, L65A-NDM-1, and T94A-NDM-1 as the initial structures were used in MD simulation. The MM-GBSA calculation predicted that the binding of F46A, L65A, and T94A to carnosic acid was weaker than WT-CA (F46A was −0.38 kcal/mol, L65A was −2.86 kcal/mol, and T94A was −1.77 kcal/mol), as shown in **Table 4**.

**Table 4:** The binding free energy (kcal/mol) and binding constants of the WT-CA, F46A-CA, L65A-CA, and T94A-CA systems based on the fluorescence spectroscopy quenching method.

	WT-CA	F46A-CA	L65A-CA	T94A-CA
Δ*G*_bind_ (kcal/mol)	−12.16	−0.38	−2.86	−1.77
Binding constants K ml·µg^−1^	0.45644	0.12715	0.33597	0.32911

Furthermore, the interactions between carnosic acid and WT-NDM-1, F46A-NDM-1, L65A-NDM-1, and T94A-NDM-1 were investigated by fluorescence quenching. According to experimental results, the linear fitting plots of *r*/*D_f_* vs. *r* between carnosic acid and WT-NDM-1, F46A-NDM-1, L65A-NDM-1, and T94A-NDM-1 can be made in **Figure 5D** and based on the plots the value of the binding constants, *K*, can be obtained and the binding constants were 0.45644, 0.12715, 0.33597, and 0.32911 ml·*µg*^−1^ for WT-NDM-1, F46A-NDM-1, L65A-NDM-1, and T94A-NDM-1. In **Table 4**, the binding constants of the interaction between carnosic acid and proteins decrease in the following order: WT > L65A > T94A > F46A at 300 K. It indicated that the binding of WT-NDM-1 with carnosic acid is stronger than those of mutants. The experimental results and theoretical results are in agreement (**Table 4**). Therefore, the residues of Phe46, Tyr64, Leu65, Asp66, and Thr94 played crucial roles in the binding of carnosic acid to NDM-1. Interestingly, residues of Phe46, Tyr64, Leu65, Asp66, and Thr94 are not located in the catalytical active region of NDM-1, which is binding sites of the substrate. This result implied that the inhibitory mechanism of carnosic acid against NDM-1 is not the competition with the substrate of NDM-1.

Subsequently, the inhibitory activity of carnosic acid against mutants was tested by the enzyme inhibition assays. In **Figure 5E**, the carnosic acid has no obvious effect on the activity of mutants. Moreover, the steady-state kinetics of the bioactivity of NDM-1 (wild type or mutants) treated with carnosic acid were shown in **Figure 5F**. As shown in **Figure 5F**, the reaction rates are 0.00742, 0.0225, 0.02164, and 0.02277 for WT-NDM-1, F46A, L65A, and T94A, respectively. These results shown that the bioactivity of mutants treated with carnosic acid has no obviously change compared with free WT-NDM-1, while the reaction rate of WT-NDM-1 decreased sharply when treated with carnosic acid. These results are consistent with the thermodynamic results (**Figure 5E**), implying that carnosic acid has no obvious effect on the activity of mutants. The findings indicate that due to the mutation of residues, the binding affinity of carnosic acid with NDM-1 decreased, resulting in a loss of inhibitory activity. Therefore, the 3D structure of NDM-1-carnosic acid complex is reliable by MD simulation.

**Figure 6:**
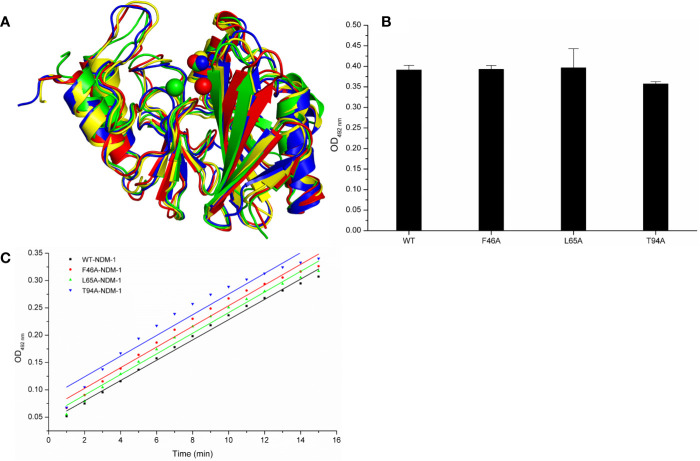
The comparison of characterization between WT-protein and mutants. **(A)** The overlap stable structures of WT-protein (red), F46A (green), L65A (blue) and T94A (yellow); **(B)** The hydrolysis activities of WT-protein or mutants; **(C)** The steady-state kinetics of the hydrolysis activity of WT-NDM-1 and mutants.

Meanwhile, the 100-ns molecular dynamics simulations were performed for the mutants. In **Figure 6A**, there was no significant change of the conformation between WT-protein and mutants through the molecular modeling. The binding sites of Zn^2+^ in the mutants were very similar to those of the WT-protein. In addition, the activities of WT-NDM-1 and its mutants were further determined using the same concentration. As expected, the mutated protein activity value deviation was within 10% compared with WT-NDM-1 (**Figure 6B**), suggesting that mutation of any of these residues did not affect the activity of NDM-1. In addition, the steady-state kinetics of WT-NDM-1 and mutants were provided. As shown in **Figure 6C**, the reaction rates are 0.01855, 0.0189, 0.01887, and 0.01891 for WT-NDM-1, F46A, L65A, and T94A, respectively. These results shown that the bioactivity of mutants has no obviously change compared with WT-NDM-1. Together, these results indicated that no significantly influence on structure or activity was observed for the site-directed mutation of F46A, L65A or T94A in NDM-1.

### Analysis of Inhibition Mechanism

**Figure 7:**
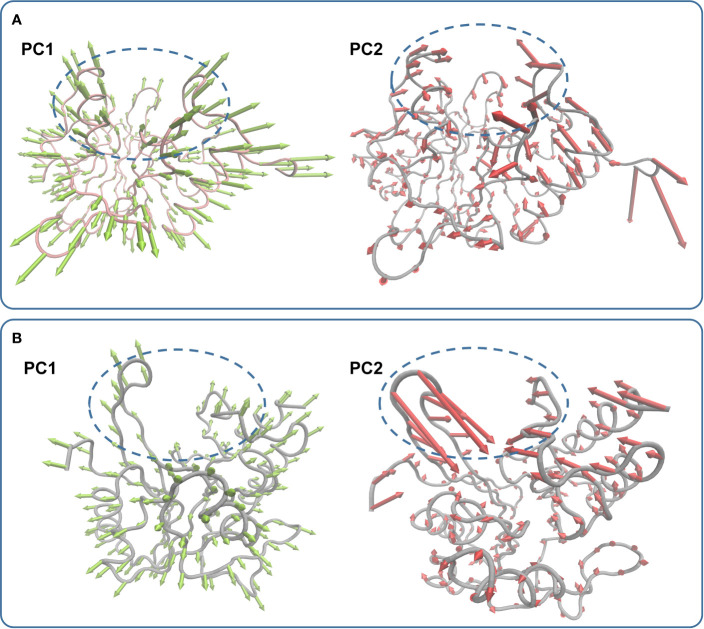
Principal component analysis based on the simulation trajectory. The first and second principal components (PC1 and PC2) in the NDM-1-CA complex **(A)** and free NDM-1 **(B)** obtained by PCA are depicted as cones on the Cα. The length of the cones represents the size of the movement.

In the simulation, carnosic acid inhibited protein activity mainly by affecting the active region of NDM-1. Subsequently, the key movements of NDM-1 containing or not containing carnosic acid was explored by Principal Component Analysis (PCA) of the NDM-1-CA compound system. In **Figure 7A**, the active region was observed to have significant extensional motion to Zn^2+^ in the first element (PC1) and the second element (PC2) in the NDM-1-CA complex system. In addition, amino acid movement near the active region was obvious. Nevertheless, these two forms of motion were significantly impaired in PC1 and PC2 of free NDM-1 system in **Figure 7B**.

**Figure 8:**
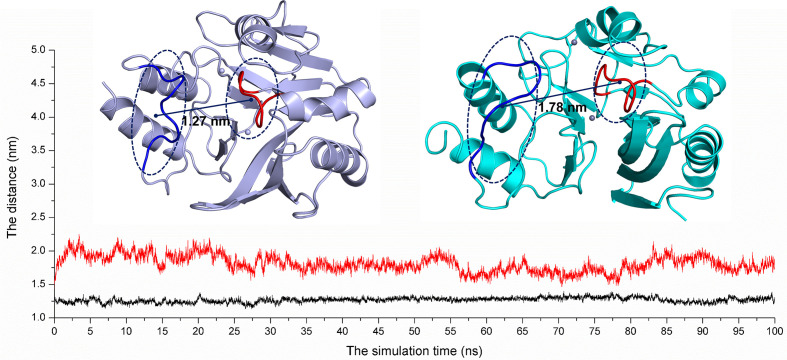
The average distances from Thr119-Met126 to Ser217-Asp225 were calculated for the free NDM-1 system (black line) and the NDM-1-CA complex system (red line).

On the other hand, according to reports in literature (Guo et al., 2011), the NDM-1 active site was located in the loop region between Thr119-Met126 and Ser217-Asp225. Therefore, the change in the active region can be judged by calculating the loop region distance. As shown in **Figure 8**, the average distance from Thr119-Met126 to Ser217-Asp225 in the free NDM-1 system was 1.27 nm. However, in the complex system, the average distance of Thr119-Met126 to Ser217-Asp225 was 1.78 nm, respectively. Therefore, it was significantly different compared to the distance in the free system. This conformation changes of the active region of NDM-1 make the binding affinity of substrate with NDM-1 weaker, resulting in the catalytic activity loss of NDM-1. Thus, it can be seen residues of Phe46, Tyr64, Leu65, Asp66, and Thr94 are the allosteric sites of NDM-1, and carnosic acid is the novel allosteric inhibitor target NDM-1.

In 2013, Sohier et al. (2013) found that camelid nanobody can inhibit VIM metallo-β-lactamases. The inhibitor mainly bound to Loop 6 and the end of the α2 helix of VIM-4. The binding site of the complex was far from the active site, but it can change the substrate binding and catalytic properties of VIM-4. The residues of T107YVF110 contributed the major binding affinity for the ligand binding with the protein. In addition, DNA aptamer can be used as an allosteric inhibitor to bind Loop 4 and Loop 6 of 5/B/6 metallo-β-lactamase (Khan et al., 2019). In the complex, the binding sites of residues were Thr76, Lys78, Phe103, Lys104, Lys107, and Tyr208. In our work, the NDM-1-CA complex allosteric sites Phe46, Tyr64, Leu65, Asp66, and Thr94 were mainly located in L3 and α1 helix, which are obviously different with the results of the previous literatures (Sohier et al., 2013; Khan et al., 2019; **Supplementary Figure S1**). Therefore, we believed that the binding site of the NDM-1-CA complex is the novel allosteric site of NDM-1.

## Discussion

At present, the combination of antibiotics with β-lactamase inhibitors is an effective method for improving antibacterial activity (Everett et al., 2018). The inhibitors are comprised of chelates (Falconer et al., 2015), mildew products (Zhang et al., 2017), and analogs of chemicals (Yarlagadda et al., 2018). Venkateswarlu (Yarlagadda et al., 2018) established that vancomycin analogues restored meropenem activity against gram-negative pathogens. The inhibitor can penetrate the outer membrane of GNP and inactivate the enzyme by depleting metal ions (Zn^2+^). However, many of these drugs are chemically synthesized and have not undergone clinical trials. Furthermore, these inhibitors are competitive inhibitors, which may contribute to the multidrug resistance. Therefore, the discovery of natural allosteric inhibitors that work against β-lactamase is the current need.

In this study, based on the virtual screening approach, we found that carnosic acid exhibited an inhibitory effect on NDM-1. Currently, carnosic acid has important applications in the fields of medicine, food, and cosmetics (Bauer et al., 2012). Scholars have revealed that the toxicity of carnosic acid is low. In the acute toxicity study, the oral lethal dose of mice was greater than 7000 mg/kg (Wang et al., 2012). It is noteworthy that food grade carnosic acid has appeared on the market (Masuda et al., 2002). At the same time, sources of carnosic acid are widely available, and it is convenient to manufacture (Birtic et al., 2015). Therefore, it has potential advantages as a clinical application inhibitor. It was established by MIC and time-killing assays that carnosic acid can restore the antibacterial activity of meropenem and inhibit NDM-1. We speculated that carnosic acid can improve the antibacterial ability of meropenem by inhibiting NDM-1. Contrary to other reported inhibitors, the results obtained from molecular modeling show that carnosic acid bound to NDM-1 *via* strong interaction with residues of Phe46, Tyr64, Leu65, Asp66, and Thr94. However, these residues are not in the active pocket of protein, implying that residues of Phe46, Tyr64, Leu65, Asp66, and Thr94 are the allosteric sites of NDM-1. Due to the binding of carnosic acid to the allosteric sites, the conformation of the active sites of NDM-1 was altered, leading to the loss of bioactivity. Simultaneously, the fluorescence experiments confirmed this hypothesis. Therefore, these studies have contributed to the development and application of NDM-1 inhibitors.

## Data Availability Statement

The original contributions presented in the study are included in the article/**Supplementary Material**; further inquiries can be directed to the corresponding authors.

## Author Contributions

XN and JW designed the experiment. YY and YGu wrote the paper and conducted experiments. YZ and YGa assisted in the completion of the experiment. XW conducted data collection. All authors contributed to the article and approved the submitted version.

## Funding

The authors acknowledge the financial support from the National Nature Science Foundation of China [Grant no. 31872525 and 81861138046].

## Conflict of Interest

The authors declare that the research was conducted in the absence of any commercial or financial relationships that could be construed as a potential conflict of interest.
