# Supplementary material for: Discovery of a Novel Natural Allosteric Inhibitor That Targets NDM-1 Against Escherichia coli
Source: Front Pharmacol. 2020 Oct 2;11:581001. doi: 10.3389/fphar.2020.581001 (PMC7566295; doi:10.3389/fphar.2020.581001)
Supplement: Supplementary file 1 [file DataSheet_1.doc]

**
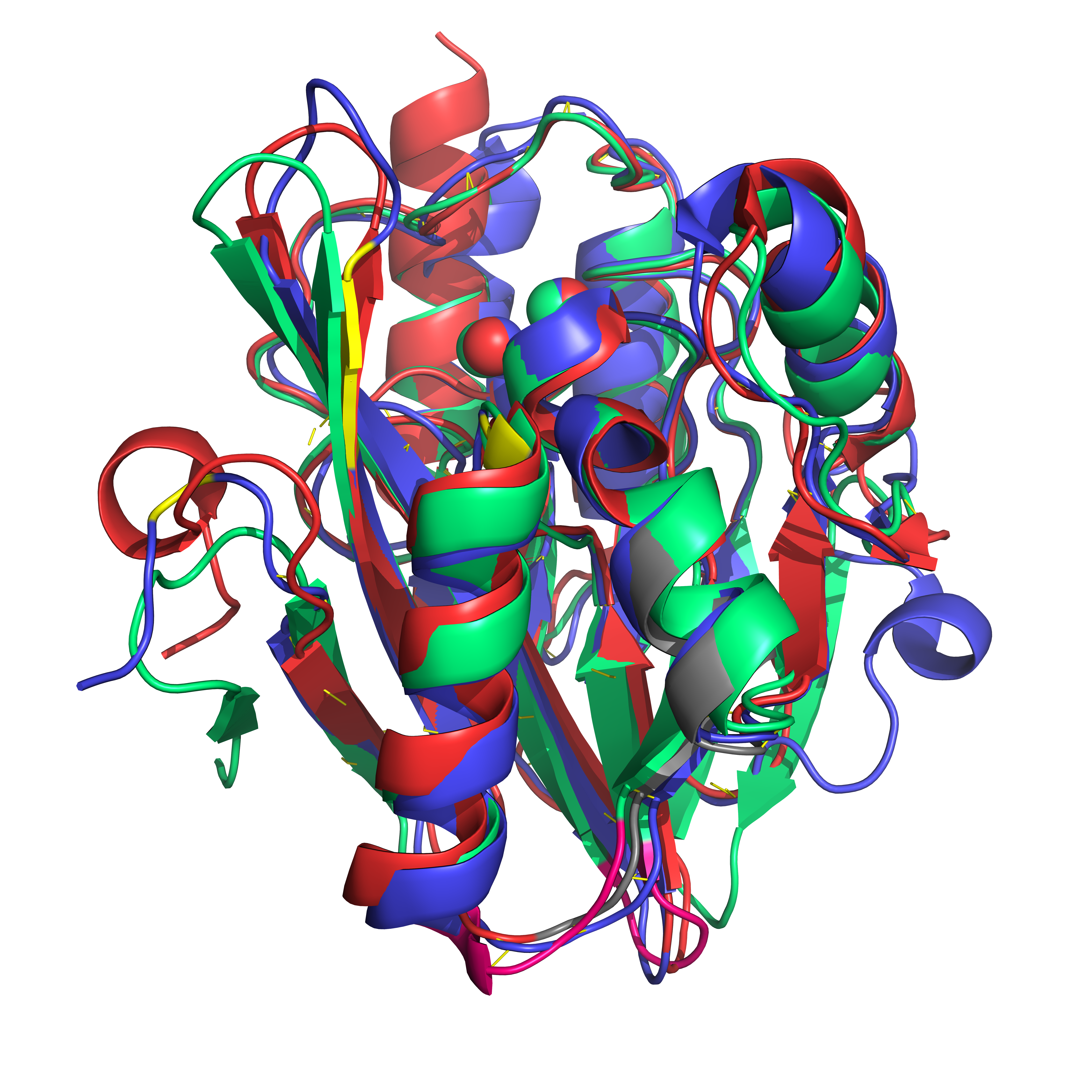
**

**Figure S1 X-ray crystal structure of Metallo-β-lactamases.** The NDM-1 is blue and allosteric site is yellow; the 5/B/6 metallo-β-lactamase is green and allosteric site is magenta; the VIM-4 is red and allosteric site is gray.
